# Homœopathic Globules

**Published:** 1865-05

**Authors:** 


*Homœopathic Globules.*—Two children have been brought up at the Wisbech Police Court, charged with stealing several bottles of homœopathic medicine from the shop of Mr. Finnell. It was said in court that they had eaten the contents of more than 20 bottles without "being either better or worse for it." The children were dismissed with a reprimand. —*London Lancet.*

